# Effect of malaria on HIV/AIDS transmission and progression

**DOI:** 10.1186/1756-3305-6-18

**Published:** 2013-01-17

**Authors:** Abebe Alemu, Yitayal Shiferaw, Zelalem Addis, Biniam Mathewos, Wubet Birhan

**Affiliations:** 1 Department of Medical Parasitology, School of Biomedical and Laboratory Sciences, College of Medicine and Health Sciences, University of Gondar, Gondar, Ethiopia; 2 Department of Medical Microbiology, School of Biomedical and Laboratory Sciences, College of Medicine and Health Sciences, University of Gondar, Gondar, Ethiopia; 3 Department of Immunology and Molecular Biology, School of Biomedical and Laboratory Sciences, College of Medicine and Health Sciences, University of Gondar, Gondar, Ethiopia

**Keywords:** Malaria, HIV/AIDS, Transmission, Progression

## Abstract

Malaria and HIV are among the two most important global health problems of developing countries. They cause more than 4 million deaths a year. These two infections interact bidirectionally and synergistically with each other. HIV infection increases the risk of an increase in the severity of malaria infection and burdens of malaria, which in turn facilitates the rate of malaria transmission. Malaria infection is also associated with strong CD4+ cell activation and up-regulation of proinflammatory cytokines and it provides an ideal microenvironment for the spread of the virus among the CD4+ cells and for rapid HIV-1 replication. Additionally, malaria increases blood viral burden by different mechanisms. Therefore, high concentrations of HIV-1 RNA in the blood are predictive of disease progression, and correlate with the risk of blood-borne, vertical, and sexual transmission of the virus. Therefore, this article aims to review information about HIV malaria interactions, the effect of malaria on HIV transmission and progression and the implications related to prevention and treatment of coinfection.

## Review

### Background

Malaria and HIV are among the two most important global health problems of developing countries. They cause more than 4 million deaths a year [1]. Malaria, sometimes called the “king of diseases”, is caused by protozoan parasites of the genus *Plasmodium*. It is one of the leading causes of illness and death in the world. Nine out of ten of these deaths occur in Africa and the rest occurs in Asia and Latin America, being the world’s most prevalent vector-borne disease. It is the fourth leading cause of death of children under the age of five years and pregnant women in developing countries [2,3]. The proportion increases each year because of deteriorating health systems, growing drug and insecticide resistance, climate change and natural disasters [4,5].

HIV/AIDS is also one of the most destructive epidemics the world has ever witnessed. In 2007 an estimated 33.2 million people were living with HIV worldwide, while 2.5 million of these people were children under 15 years old. Furthermore, 420,000 children under 15 years were newly infected with HIV in 2007. Nearly 90% of all HIV-positive children live in sub-Saharan Africa. In Ethiopia, while 66% of the population is at risk of malaria, 1.5 million people are infected with HIV [6,7].

In addition to this increased prevalence in developing countries Malaria and HIV/AIDS overlap geographically, primarily in sub-Saharan Africa, Southeast Asia and South America. While infection with either malaria or HIV/AIDS can cause illness and death, infection with one can make infection with the other worse and/or more difficult to treat. The two diseases have particularly devastating effects for those living in malaria endemic regions throughout the world. Pregnant women suffer particularly serious consequences when infected with both HIV/AIDS and malaria. HIV/AIDS can increase the adverse effects of malaria, including anemia and placental malaria infection [8].

These two infections interact bidirectionally and synergistically with each other. HIV infection can increase the risk and severity of malaria infection and the increased parasite burdens might facilitate higher rates of malaria transmission. Individuals in malaria-endemic areas that are considered semi-immune to malaria can also develop clinical malaria if they are infected with HIV. Also malaria infection is associated with strong CD4+ cell activation and up-regulation of proinflammatory cytokines, providing an ideal microenvironment for the spread of the virus among CD4 + cells and thus for rapid HIV-1 replication [9].

Understanding of the human immune response to malaria and HIV leads us to expect that either infection might influence the clinical course of the other. Many other types of infections are associated with at least a transient increase in HIV viral load. Hence, it is logical to expect malaria to do the same and potentially to accelerate HIV disease progression. On the other hand, the control of malaria parasitaemia is immune mediated, and this prevents most malarial infections from becoming clinically apparent in semi-immune adults in endemic areas [10]. The immune deficiency caused by HIV infection should, in theory, reduce the immune response to malaria parasitaemia and therefore increase the frequency of clinical attacks of malaria. So HIV infection affects the clinical presentation, severity and response to treatment of malaria cases. The clinical impact of these interactions varies depending on the intensity of malaria transmission in the area (and consequent level of host immunity) and the individual affected (e.g. adult, child or pregnant woman) [11]. However, in different malaria HIV co-endemic countries there has been little or no research conducted regarding this topic. The aim of this article is, therefore, to review existing information about HIV malaria interactions, the effect of malaria on HIV transmission and progression and the implications related to prevention and treatment of coinfection.

### Methods

This article was written after reviewing the pertinent information available about the interaction between HIV and malaria from Hinari and PubMed web sites (http://www.who.int/hinari/en/ and http://www.ncbi.nlm.nih.gov/pubmed). Although much has been published in the last 10 years regarding the interaction of HIV and malaria in sub-Saharan Africa and other parts, we still need more information so as to understand the issues that will help us develop effective programs in sub-Sahara Africa. Therefore, we have used 90% of the literature which is less than ten years old.

### Evidence of effect of malaria on HIV/AIDS transmission and progression

On average, without treatment, a person progresses from infection to AIDS in about seven to ten years. This is a rough estimate that varies somewhat depending on how the patient contracted HIV. In general, the rate of progression seems to be higher for those who are infected through blood contact, rather than through sex. Transfusion recipients may be the fastest to progress, on average in about seven years. Hemophiliacs and injection drug users are next fastest. Homosexual men tend to progress a little more slowly. These are generalizations, of course, and an individual’s rate of progression may be quite different [12].

HIV disease progression and transmission are strongly associated with blood viral burden. An increased concentration of human immunodeficiency virus type-1 (HIV-1) has been observed in the blood of men in Malawi relative to men in the USA and Europe, independent of CD4+ cell count. Although the problem is multi factorial, high levels of viral replication and blood viral burden could provide one explanation for the scope and magnitude of the HIV epidemic in sub-Saharan Africa. So, high concentrations of HIV-1 RNA in the blood are predictive of disease progression, and correlate with the risk of blood-borne, vertical, and sexual transmission of the virus. Accordingly, understanding the factor(s) that increase the HIV viral burden is critical to patient management and efforts towards HIV prevention [13,14].

Malaria is an important cause of disease in HIV-infected adults wherever the two infections coexist. The impact of malaria on HIV-1 is less clear. Efficient reverse transcription and integration of the HIV genome into the host DNA does not occur until the immune cells are activated [15]. Because malaria infection is associated with strong CD4+ cell activation and up-regulation of proinflammatory cytokines, it provides an ideal microenvironment for the spread of the virus among the CD4+ cells and thus for rapid HIV-1 replication. This has been described for malaria in an *in vitro* model [16]. *In vivo*, HIV-1 viral load first increases in malaria infected patients and then partially decreases 4 weeks after anti-malaria treatment [17].

In Malawi, blood concentrations of HIV-1 were seven-fold higher in HIV-1-infected adults with acute uncomplicated malaria than in HIV-1-infected blood donors without malaria. As with other acute infections, increased viral burden was reversed by effective malaria treatment. These findings accord with results of *in vitro* studies, in which HIV-1 replication was increased 10–100 fold in peripheral blood mononuclear cells exposed to malaria antigens or malaria pigment, mediated by enhanced expression of the cytokine tumor necrosis factor alpha (TNF-α) [18].

Some authorities have postulated that much of the improved survival seen in HIV-infected adults and children with use of daily cotrimoxazole prophylaxis is due to a reduction in malaria infection [19]. In cotrimoxazole prophylaxis studies performed in malaria endemic regions, malaria is consistently reduced. Studies have also demonstrated that a transient increase in HIV plasma viral load occurs during a malarial fever episode. However, the convalescent viral loads were similar to levels prior to the disease event, suggesting that these events do not modify the individual viral load significantly [19].

Another study also showed that acute malaria is associated with an increase in viral load and a decrease in CD4+ cell count, but this generally returns to pre-infection levels a few weeks after successful malaria therapy. This could theoretically lead to increased HIV transmission or more rapid disease progression, but has not been demonstrated prospectively. Therefore, studies on the effect of malaria on the burden of HIV in the blood, show concentrations of HIV-1- RNA in the blood were higher in HIV-infected adults with acute malaria illness than in HIV-infected adults who were aparasitaemic, with viral load decreasing over time, probably due to the treatment of malaria [20].

A small study compared the plasma viral load in a group of HIV-infected patients in Guinea-Bissau (most of whom were infected with HIV-2) between the wet and dry seasons, and noted no overall changes in mean plasma RNA. In individual patients with *Plasmodium falciparum* parasitaemia, HIV-2 viral load rose significantly, and in one patient, this episode was the only time at which plasma viremia was detectable. This study strongly suggests that clinical malaria could contribute to higher plasma viremia in HIV-infected patients (at least in the short term and possibly sustained), which has potential implications for more rapid HIV disease progression as well as an increased likelihood of HIV transmission [21].

Cohort studies in Cameroon showed that malaria infection during pregnancy may increase the risk of mother-to-child transmission of HIV. One potential mechanism for this was evaluated *in vitro*, where binding of recombinant *P*. *falciparum* adhesin to chondroitin sulfate A on human placental cells increased HIV-1 replication in those cells, possibly via TNF-α stimulation [22].

Other study in Gambia showed that among HIV-infected pregnant women, placental histopathologic malaria was associated with higher concentrations of both peripheral and placental HIV-1 RNA. After adjusting for confounders, women with placental histopathologic malaria had a 1.7-fold higher peripheral HIV-1 viral load and a 2.0-fold higher placental HIV-1 viral load than those without placental malaria. Although peripheral and placental blood film microscopy had a low sensitivity for detecting malaria infection, peripheral and placental parasitemia were also associated with higher placental HIV-1 viral load. Among women with placental parasitemia, placental parasite density was positively correlated with both placental and peripheral HIV-1 viral load [23].

#### Mechanisms of how malaria affects HIV transmission and progression

##### Immunological mechanisms

Scientists speculate that malaria-induced changes in viral load result from “a series of immunological mechanisms” [19]. For example, *P*. *falciparum* infection might promote macrophages and CD4+ cells to activate viral transcription. A high parasite density likely elicits a strong immune response, leading to a high turnover of HIV-1 RNA and fever indicates a cytokine response that might raise HIV-1 RNA concentrations. A number of studies suggest more effective sexual transmission of HIV and an accelerated progression to clinical disease is seen in people with elevated concentrations of HIV-1 RNA in the blood [24].

Many intercurrent infections and even immunizations can lead to a short-term increase in plasma viral load in HIV-infected people, often accompanied by a fall in the CD4+ cell count [25]. This has been interpreted as being due to the activation of T cells by the cytokines generated in response to the infecting organisms, which in turn increases the population of susceptible cells for HIV replication. The massive out-pouring of TNF-α and other pro inflammatory cytokines in response to acute malaria infection, together with the activation of CD4+ cells, would be expected to generate ideal conditions for HIV replication, especially as TNF-α can act directly on the HIV long terminal repeat to up-regulate viral replication. *In vitro* studies have demonstrated that exposure of HIV-infected T-cell cultures to malarial antigens leads to significantly enhanced viral replication. Increased HIV replication was associated with high levels of cellular proliferation and activation and could be blocked by antibodies to TNF-α [25].

Additionally, HIV replication in CD4+ cells requires that cells express an activated phenotype and intercurrent infections that induce immune activation have been found to increase HIV disease progression. In large areas of the tropics, HIV and malaria coexist with considerable potential for co-infection [26]. In non-immune individuals, malaria infection leads to a pro-inflammatory (T helper 1- type) immune response with activation of CD4+, CD4 + 5RO + T cells to produce interferon gamma (IFN-γ). T cell-derived signals synergize with parasite-derived stimuli to activate monocytes and macrophages to produce interleukin-1 (IL-1) and IFN-γ. As the CD4+ CD4 + 5RO^+^ T cell subset is a preferred target for HIV replication, these would seem to be ideal conditions for rendering individuals susceptible to HIV infection and for re-activation of viral replication in infected individuals, raising the possibility that malaria infection may lead to more rapid progression to AIDS and death [27].

More than 95% of HIV-1 in the plasma is produced by infected-activated CD4+ lymphocytes [28]. *P*. *falciparum* malaria causes increased CD4+ cell activation, increasing the number of susceptible target cells for HIV-1 infection, potentially resulting in increased HIV-1 RNA levels and continued new infection of susceptible activated CD4+ cells. CD4+ cell activation associated with malaria may persist despite therapy. The elevated RNA levels observed for 4 weeks after therapy are not therefore surprising. The fact that malaria subjects with the highest CD4+ cell percentages at enrollment experienced the largest RNA declines is also consistent with the hypothesis that CD4+ cell activation is partly responsible for raised HIV RNA levels. Alternatively, malaria patients with higher CD4+ cell percentages may be better able to control either infection, resulting in a more rapid decline in RNA [28].

Increases in HIV-1 RNA related to malaria might be mediated through a series of immunological mechanisms. *P*. *falciparum* infection might promote macrophages and CD4+ cells to activate viral transcription. These mechanisms could explain the differences observed between the groups classified according to parasitaemia, fever, and CD4+ count in some studies. High parasite density is likely to elicit strong immune activation, leading to a high turnover of HIV-1 RNA. Fever indicates a cytokine response that might increase concentrations of HIV-1 RNA. The modest increase in HIV-1 RNA concentration among people with lower CD4+ counts and higher concentrations HIV-1 RNA at baseline might indicate poor CD4+ cell function, which restricts the otherwise normal response to the cytokine stimuli of malaria, leading to a reduced increase in HIV-1 RNA turnover [29].

However, conflicting results have been obtained from *in vitro* studies. Firstly, an increase in HIV replication in blood mononuclear cells exposed to either malaria antigens or malaria pigment has been observed, and this was shown to be associated with increased production of TNF-α. Subsequently, it was reported that short stimulations with *P*. *falciparum* antigens down-regulated CCR5 but not CXCR4 [30]. However, longer stimulation up-regulated CCR5 through the induction of IFN-γ and this IFN-γ production blocked HIV replication. More recently, mononuclear cell activation by malaria antigens was shown to render these cells more susceptible to HIV infection and to reactivate replication of endogenous HIV in cells from HIV-infected adults. Differences in viral strain, host cell type, and culture conditions might account for these conflicting reports [31].

During acute malaria, approximately 10% of the HIV-1 pool was derived from CD14+ macrophages, and this is likely to be an underestimate in view of the limitations in efficiency inherent in immunomagnetic capture [32]. This effect of malaria coinfection is similar to the effect of tuberculosis on HIV-1 replication and is supported by the finding in an HIV transgenic mouse model that murine malaria markedly up regulated proviral transcription, predominantly within antigen-presenting cells [32]. Macrophages are long-lived, migratory reservoirs of HIV-1 infection that disseminate virus to lymphocytes during cell–cell interactions. These cells may, therefore, have a sustained effect on viral propagation and dissemination, thereby promoting disease progression. HIV-1 replicating within macrophages may also be less accessible to antiretroviral drugs.

In addition, the effect of co-infection with malaria and HIV on dendritic cells has not yet been extensively investigated. Dendritic cells (DC) have an important role in the induction and maintenance of cellular anti-HIV immunity [33]. Strong polyspecific CD8+ T cell and sustained CD4+ T cell responses have been associated with protection against HIV. As with any infection, the initiation of an immune response depends on the presentation of viral antigens by DC to T cells [33]. DCs can present the virus either endogenously when infected, or can cross-present it after the capture of viral antigens from infected or apoptotic cells and several studies have suggested that malaria infection can impair human DC function *in vitro*. Moreover, in a recent report using rodent malaria, DCs from infected animals were shown have impaired cross-presentation activity [34]. It is therefore possible that this inhibitory effect might influence the establishment and/or maintenance of HIV anti-immunity.

##### Malaria affects the peripheral circulation leukocyte subsets

Normal peripheral blood is composed of three main cellular elements: Red blood cells (erythrocytes), White blood cells (Leucocytes), and platelets (Thrombocytes). Malaria infection is known to induce apoptosis, the mean percentage of spontaneous apoptosis of mononuclear cells was found to be higher in patients with acute as well as chronic asymptomatic *P*. *falciparum* infection, compared to age and sex matched controls [35]. Thus parasite-induced apoptosis would contribute to reducing the immune response directed towards critical antigens, by increasing the fragility of potential effectors cells. Therefore, besides the cytokines (TNF-α and IFN-γ) and malaria antigen mediated activation-induced sequestration of the lymphocyte in the lymph nodes, which then causes depletion of these cells in the peripheral blood, malaria antigen-induced apoptosis could also play a role in altering the lymphocyte composition in the peripheral blood during malaria infections [35].

In Children with cerebral or uncomplicated malaria, the frequency and absolute number of peripheral T cells was also lower than normal and the degree of disease induced T cell outflow from the peripheral blood was correlated with disease severity. Studies have reported lower total leukocyte and lymphocyte counts but a high number of activated cells in malaria patients with a distinct pattern observed between *P*. *falciparum* and *P*. *vivax* infections. Furthermore, in studies done in children aged 3–6 years who were infected with *P*. *falciparum*, lower CD4+ and CD8+ cell counts were observed in those with acute malaria when compared with children with no parasitemia or in those with asymptomatic parasitemia [36].

Regulation of CD8+ T cell responses during malaria infection is also common. The suppression of CD8+ T cell responses during blood stage infection has wider consequences for the immune response not only to malaria but also to other infectious agents. Indeed, the suppression of CD8 T cell responses is consistent with the association of Burkitt’s lymphoma and endemic malaria. It has been suggested that malaria must induce loss of control of Epstein Bar Virus (EBV) infected B lymphocytes or provide a mitogen or mechanism that stimulates B cell growth. The dysregulation of CD8+ T cells as a result of malaria infection might be even more extensive. Suppression of specific CD8+ T cell responses during blood stage malaria may explain the higher viral load in HIV individuals not only during but after infection with *P*. *falciparum*[37].

##### Malaria causes severe anemia

Inadequate prevention and control of malaria leads to excess malaria associated severe anemia, which often requires blood transfusion. Although identified repeatedly as an important public health measure, blood safety remains poorly documented in many sub-Saharan African countries, and inadequate HIV-1 screening is likely to remain a widespread problem [38]. Also pregnant women with both malaria and HIV-1 are at higher risk of developing severe anemia than are women with either infection alone, and also have a higher risk of delivering a premature or low birth weight infant. With the risk of transmission of HIV-1, the use of blood transfusion in the management of severe pediatric anemia has become an important clinical decision problem in Africa [38].

The prevalence of HIV infection among blood donors has increased in most developing countries, and at least 10% of all African pediatric acquired immunodeficiency syndrome (AIDS) cases may have arisen from contaminated blood transfusions. Many African countries, particularly where *P*. *falciparum* malaria is endemic and HIV/AIDS is a major health issue, cannot maintain an adequate blood supply, and fail to screen all their donated blood. Even screened blood can be infectious, with a risk that depends on the background sero prevalence among the blood donors and on the quality of the screening [39].

#### Effect of malaria on mother to child transmission of HIV

Mother-to-child transmission (MTCT) of HIV occurs in an estimated 500,000 newborns and infants each year in Sub-Saharan Africa [40]. Pregnant women infected with HIV are often co-infected with malaria, and various blood-borne and intestinal helminth infections in Sub- Saharan Africa. These co-infections may increase the risk for MTCT of HIV. Thus the eradication of this coinfection in pregnant women could reduce MTCT of HIV. Several observations have implicated malaria as a potential risk factor for MTCT of HIV. Malaria infections can increase HIV loads in peripheral blood and greater viral loads enhance the risk for MTCT of HIV [40].

On the other hand, *P*. *falciparum* has been shown to stimulate HIV-1 replication through the production of cytokines (IL-6 and TNF-α) by activated lymphocytes [41]. *Plasmodium falciparum* also increases the potential reservoir for HIV in the placenta by increasing the number of CCR5^+^ macrophages. So, the adverse effects of HIV–*Plasmodium* co-infection can, in the most part, be attributed to immunological interactions. Not only is the immune-mediated control of malaria attenuated by HIV-mediated damage to the immune system, but malaria itself can cause T-cell activation and cytokine release that can stimulate HIV replication. Immunity is also altered during pregnancy and might account for some of the detrimental effects seen in co-infected pregnant women [41].

Placental HIV-1 viral load is increased in women with placental malaria, especially those with high parasite densities. However, the effect of malaria on mother-to-child transmission of HIV is unclear because published studies to date have given conflicting findings [42]. It has been suggested that the discrepancy might be due to variations in maternal immunocompetence. That is, immunocompromised mothers have deranged chemokine and cytokine profiles, less protective immune responses, and consequently higher parasite densities and viral loads, leading to an increased risk of MTCT of HIV. If HIV-positive mothers co-infected with placental malaria are at an increased risk of transmitting HIV to their infants, malaria prophylaxis during pregnancy becomes an urgent priority, not only because of the adverse impact of malaria infection during pregnancy, but because co-infection could potentially increase the risk of vertical transmission of HIV [42,43].

Research investigating the impact of malaria infection on the risk of MTCT of HIV has reported conflicting results. In a study from Uganda, placental malaria was associated with an increased risk of MTCT (61). However, no association between placental malaria and risk of MTCT was detected in Mombasa, Kenya [14]. Differences between the studies in maternal immunological status, plasma viral load, HIV subtype or mode of delivery may account for the inconsistent findings. These conflicting results may also reflect the complex relationship between maternal immune responses to malaria which, depending on the immune response, may either have a protective effect or increase the risk of MTCT. The direction of effect may depend on the degree of HIV related immunosuppression and on the severity of malaria and thus the degree of placental monocytes infiltrates and proinflammatory cytokine and chemokine responses [44].

Several mechanisms have been identified by which placental malaria (PM) may affect MTCT of HIV-1. For example, malarial infection up-regulates HIV-1 CCR5 chemokine co-receptor expression on placental macrophages, and increase placental viral loads, thereby increasing the risk of HIV-1 transmission. Additionally, PM infection and subsequent inflammatory responses may damage the integrity of the placenta and increase MTCT of HIV-1 [45]. Although another study in Zimbabwe noted that PM was associated with monocytic infiltration in the deciduas of placental floor, with no associations noted with infiltration at other placental sites, and in general infiltration did not modify the relation between PM and MTCT. Malaria may also affect MTCT of HIV-1 indirectly, through its adverse effects on maternal and infant immune function and pregnancy outcomes (e.g., low birth weight) [46].

Malaria infection in the placenta was also shown to increase RNA viral load. In particular, *in vitro* haemozoin treatment of intervillous and peripheral blood mononuclear cells increased RNA viral load and induced the secretion of IFN-γ and TNF-α, two cytokines that are detrimental during pregnancy. Increased expression of the chemokine receptor CCR5, the main co-receptor for mother-to-child HIV transmission, has been observed on maternal placental macrophages during malarial infection [47]. This suggests that there may be an increase in the rate of mother-to-child transmission, especially in conjunction with increased viral load. However, contradictory results have been obtained [48]. These results might have been confounded by the fact that malaria infection also induces the production of chemokines like MIP-1 alpha and beta which can bind to the receptor CCR5, and are potent inhibitors of HIV invasion and could thus inhibit mother-to-child transmission [48].

In general a heightened risk of HIV transmission with placental malaria could be the result of one or more factors. There may be a disruption of the placental cellular architecture that allows an intermingling of maternal and fetal blood [49]. Another mechanism might be that placental malaria stimulates a local increase of HIV-infected macrophages and other lymphocytes, and this increases the risk of viral transmission. Alternatively, placental malaria may simply be a consequence of advanced HIV infection and higher viral load, itself associated with mother-to-child transmission. A study of maternal HIV infection and infant mortality in Malawi showed evidence for increased mortality due to placental malaria infection [50].

In contrast, recent studies suggest that selected cytokines and hormones potentially affect HIV-1 trans-placental transmission and that both innate and acquired protection play a role in MTCT [51]. First, malaria is known to induce disequilibrium in the balance between Th1 and Th2 responses, favoring the Th1 pathway. T helper responses are known to control HIV replication; hence, inducing a Th1 response in the placental compartment could lead to reduced HIV-1 replication [51].

Indeed, a moderate increase was found in the Th1 cytokine IFN-γ response in the intervillous blood mononuclear cell responses of HIV-positive mothers with placental malaria as compared to HIV-positive mothers without placental malaria [52]. Second, leukemia inhibitory factor (LIF) induces a potent inhibition of HIV replication, and this cytokine is up regulated in placentas of women who do not transmit HIV [52]. Malarial antigens may induce production of LIF that results in reduced rates of perinatal MTCT. Third, malarial antigen may result in altered chemokine production, which in turn can block chemokine receptors necessary for cellular HIV entry [52].

## Conclusion

In conclusion, evidence from studies in different countries show that; Malaria and HIV are two of the most important infectious diseases, which affect millions of people across overlapping geographic distributions. Malaria infection is associated with strong CD4+ cell activation and up-regulation of proinflammatory cytokines and it provides an ideal microenvironment for the spread of the virus among the CD4+ cells and for rapid HIV-1 replication. Additionally, malaria increases blood viral burden by different mechanisms. Therefore, high concentrations of HIV-1 RNA in the blood are predictive of disease progression, and correlate with the risk of blood-borne, vertical, and sexual transmission of the virus. Therefore, knowing of interaction between malaria and HIV are important for management or control of these diseases and further research activities in the area are highly required. Additionally, interaction between malaria and HIV during pregnancy and their putative impact on MTCT of HIV is complex and also needs further study.

## Competing interests

All authors declare that they have no competing financial or any other interest in relation to their work.

## Authors’ contributions

AA: Conceived the paper, took the lead in conception and design, and led the drafting of the paper. YS, ZA, BM, WB: contributed significantly to the writing of the paper. All authors have read and approved the final version of the paper.
